# Comparative Genetic Structure of *Cannabis sativa* Including Federally Produced, Wild Collected, and Cultivated Samples

**DOI:** 10.3389/fpls.2021.675770

**Published:** 2021-09-29

**Authors:** Anna L. Schwabe, Connor J. Hansen, Richard M. Hyslop, Mitchell E. McGlaughlin

**Affiliations:** ^1^School of Biological Sciences, University of Northern Colorado, Greeley, CO, United States; ^2^Department of Chemistry and Biochemistry, University of Northern Colorado, Greeley, CO, United States

**Keywords:** *Cannabis sativa*, NIDA, genotype, marijuana, microsatellite, phenotype, strains, hemp (*Cannabis sativa* L.)

## Abstract

Currently in the United States, the sole licensed facility to cultivate *Cannabis sativa* L. for research purposes is the University of Mississippi, which is funded by the National Institute on Drug Abuse (NIDA). Studies researching *Cannabis* flower consumption rely on NIDA-supplied “research grade marijuana.” Previous research found that cannabinoid levels of NIDA-supplied *Cannabis* do not align with commercially available *Cannabis*. We sought to investigate the genetic identity of *Cannabis* supplied by NIDA relative to common categories within the species. This is the first genetic study to include “research grade marijuana” from NIDA. Samples (49) were assigned as Wild Hemp (feral; 6) and Cultivated Hemp (3), NIDA (2), CBD drug type (3), and high THC drug type subdivided into Sativa (11), Hybrid (14), and Indica (10). Ten microsatellites targeting neutral non-coding regions were used. Clustering and genetic distance analyses support a division between hemp and drug-type *Cannabis*. All hemp samples clustered genetically, but no clear distinction of Sativa, Hybrid, and Indica subcategories within retail marijuana samples was found. Interestingly, the two analyzed “research grade marijuana” samples obtained from NIDA were genetically distinct from most drug-type *Cannabis* available from retail dispensaries. Although the sample size was small, “research grade marijuana” provided for research is genetically distinct from most retail drug-type *Cannabis* that patients and patrons are consuming.

## Introduction

Humans have a long history with *Cannabis sativa*, with evidence of cultivation dating back as far as 10,000years (Abel, 2013). The World Health Organization reports *Cannabis* as the most widely cultivated, trafficked and abused illicit drug, and it constitutes over half of worldwide drug seizures (World Health Organization, 2018). The United States is currently experiencing drastic changes in patterns of *Cannabis* use associated with widespread relaxation of laws that previously limited both medical and recreational consumption (Cousijn et al., 2018), as well as hemp cultivation. This has led to a need for extensive research into the basic biology and taxonomy of *Cannabis sativa* (Hillig, 2005; Clarke and Merlin, 2013; Lynch et al., 2016; Vergara et al., 2016; Small, 2017).

*Cannabis sativa* is the only described species in the genus *Cannabis* (Cannabaceae) but there are several commonly described subcategories that are widely recognized. There are two primary groups, which are well-supported by genetic analyses (Sawler et al., 2015; Lynch et al., 2016; Dufresnes et al., 2017; Soler et al., 2017): (1) hemp or hemp type which is legally defined in the United States as *Cannabis* containing no more than 0.3% THC, and (2) marijuana, drug type, or drug type which encompasses all *Cannabis* with THC concentrations >0.3% THC. The term marijuana is controversial, so unless referencing “research grade marijuana” as defined by the US government, we utilize the term “drug type,” as there is no acceptable widely used term for *Cannabis* that does not classify as hemp. It is important to note that much of the confusion around *Cannabis* groups is related to the fact that hemp and drug types are distinguished based on % THC content, which is a variable trait that has been selected for or against in the two groups. Hemp types tend to have higher concentrations of CBD than drug types (de Meijer et al., 1992). High THC drug types generally contain >12% THC and average ~10–23% THC in dispensaries (Potter et al., 2008; Vergara et al., 2017; Jikomes and Zoorob, 2018). Within the two major groups, *Cannabis* can be further divided into varietals or strains. High THC drug types are often categorized further in the commercial marketplace: Sativa, Indica, and Hybrid strains, which reportedly have different intoxicating effects (Heilig, 2011; Hazekamp and Fischedick, 2012; Smith, 2012; McPartland, 2017; Leafly, 2018). There is continuing debate among experts surrounding the appropriate taxonomic treatment of *Cannabis* groups, which is confounded by colloquial usage of these terms vs. what researchers suggest is more appropriate nomenclature (Small et al., 1976; Emboden, 1977, 1981; Clarke and Merlin, 2015; Small, 2015, 2016; McPartland, 2017; McPartland and Guy, 2017). Genetic analyses have not shown clear and consistent differentiation among the three commonly described high THC drug strain categories (Sawler et al., 2015; Lynch et al., 2016), but both the recreational and medical *Cannabis* communities maintain that there are distinct differences in effects between Sativa and Indica strains (Smith, 2012; Leafly, 2018).

Although *Cannabis* has been federally controlled in the United States since 1937, as of February 2021, 36 states and the District of Columbia (DC) allow regulated medical use, and 16 states and Washington DC allow adult recreational use (ProCon, 2018a). However, because the DEA lists THC as a Schedule I substance (United States Congress, 1970), research on all aspects of this plant has been limited. In the United States, a Schedule I substance is described as a drug with no accepted medical use and a high potential for abuse (United States Congress, 1970). Surgeon General Jerome Adams recently expressed concern that the current scheduling in the most restrictive category is inhibiting research on *Cannabis* as a potentially therapeutic plant (Jaeger, 2018). The University of Mississippi, funded through the National Institutes of Health/National Institute on Drug Abuse (NIH/NIDA), currently holds the only license issued by the Drug Enforcement Administration (DEA) for the cultivation of *Cannabis* for research purposes (Drug Enforcement Administration and Department of Justice, 2016). As such, NIDA serves as the sole legal provider of drug-type *Cannabis* for federally funded medical research in the United States. NIDA does not grow or distribute hemp-type *Cannabis*.

Medical research on *Cannabis* has primarily focused on isolated THC and CBD (Borgelt et al., 2013; Maa and Figi, 2014; Backes and Weil 2014; National Institute on Drug Abuse, 2016a, 2019, 2020; Baron, 2018; Citti et al., 2018; Cousijn et al., 2018) but there are hundreds of other chemical constituents in *Cannabis* (ElSohly, 2007), including cannabinoids and terpenes (Baron, 2018). Recent research has documented that NIDA-provided *Cannabis* has distinctly different cannabinoid profiles than commercially available *Cannabis* (Vergara et al., 2017). Specifically, Vergara et al. (2017) found that NIDA-reported THC and CBD concentrations were only 27 and 48%, respectively, of the mean values of commercially available drug-type *Cannabis* samples in the four US cities (Vergara et al., 2017). Due to the growing evidence that chemical constituents in various combinations and abundances in the whole plant work in concert to create the suite of reported physiological effects (Baron, 2018; Nahler et al., 2019; Russo, 2019; Ferber et al., 2020), it is important to know how strains vary in all relevant components. The chemical makeup of each variant of *Cannabis* is influenced by environmental conditions (e.g., light, water, nutrients, soil, airflow, etc.) and the underlying genetic makeup. Since genotype does not change, genetic data is essential baseline information for understanding *Cannabis* diversity, consistency, and potential effects.

In the current study, we investigated the genetic relationship of two types of NIDA-obtained *Cannabis* to commercially available drug-type *Cannabis*, as well as wild (feral) and cultivated hemp. Since *Cannabis* has been under heavy artificial selection for different traits such as THC content or industrial uses, we focused solely on genetic data. We assessed ten variable nuclear microsatellite loci targeting non-coding regions of the genome to examine genetic differentiation among our samples independent of recent human selection. Included in the present study were samples from NIDA (high THC and high THC/CBD), high THC drug type, low THC/high CBD drug type, wild growing hemp (presumed escapees from cultivation), and cultivated hemp. This study aimed to investigate where research grade *Cannabis* supplied by NIDA falls on the genetic spectrum of *Cannabis* groups.

## Materials and Methods

Cannabinoid concentrations were not measured for any of the samples, as this was a genetic study. Samples were categorized based on the information provided at the time of acquisition. A total of 49 *Cannabis* samples acquired in the United States were used in this research (Supplementary Table 1), including Wild (feral) hemp (6), Cultivated hemp (3), NIDA samples (2), high CBD drug type (3), and high THC drug type (35). The wild collected hemp was sampled from herbaria collections and is presumed to represent feral specimens that escaped from cultivation. NIDA “research grade marijuana” was limited to two samples obtained *via* another study: “high THC” defined by NIDA as containing >5–10% THC (RTI log number 13494-22, reference number SAF 027355) and “high THC/CBD” defined by NIDA as containing 5–10% of both THC and CBD (RTI log number 13784-1114-18-6, reference number SAF 027355: National Institute on Drug Abuse, 2016b). NIDA has limited the access of “research grade marijuana” for non-medical research, so we did not have access to a wider sampling of the types they provide. High THC drug-type samples were further subdivided into three frequently used colloquial strain categories: Sativa (11), Hybrid (14), and Indica (10) based on information available online (Leafly, 2018; PotGuide.com, 2018; Wikileaf, 2018; Seedfinder, 2020). *Cannabis* is genetically diverse and based on our research which included 122 samples (Schwabe and McGlaughlin, 2019), and other published research (Gao et al., 2014; Sawler et al., 2015; Lynch et al., 2016; Dufresnes et al., 2017; Soler et al., 2017; Pisupati et al., 2018), the sampling used here adequately captures the genetic diversity within and among the groups.

DNA was extracted using a CTAB extraction protocol (Doyle, 1987) modified to use 0.035–0.100g of dried flower tissue per extraction. Ten variable microsatellite loci developed by Schwabe & McGlaughlin (Schwabe and McGlaughlin, 2019) were used in this study following their previously described procedures.

GENALEX ver. 6.4.1 (Peakall and Smouse, 2006, 2012; 59, 60) was used to calculate pairwise genetic differentiation (F_ST_) and Nei’s genetic distance (D) between each of the seven groups and to determine the presence of private alleles. PCoA eigenvalues calculated in GENALEX were used to plot the PCoA in RStudio with the ggplot package (R Studio Team, 2015) with 95% confidence interval ellipses.

Genotypes were analyzed using the Bayesian cluster analysis program STRUCTURE ver. 2.4.2 (Pritchard et al., 2000). Burn-in and run-lengths of 50,000 generations were used with ten independent replicates for each STRUCTURE analysis, testing K=1–10. The number of genetic groups for the data set was determined by STRUCTURE HARVESTER (Earl and vonHoldt, 2012), which implements the method of Evanno et al. (2005).

Maverick v1.0.5 (Verity and Nichols, 2016) was used as an additional verification of Bayesian clustering analysis using thermodynamic integration to determine the appropriate number of genetic groups. The following parameters were used: admixture parameter (alpha) of 0.03 with a standard deviation (alphaPropSD) of 0.008, ten replicates (mainRepeats), 1,000 Burn-in iterations (mainBurnin), 5,000 sample iterations (mainSamples), 100 TI rungs (thermodynamicRungs), 500 TI Burn-in iterations (thermodynamicBurnin), and 1,000 TI iterations (thermodynamicSamples).

## Results

**Figure 1:**
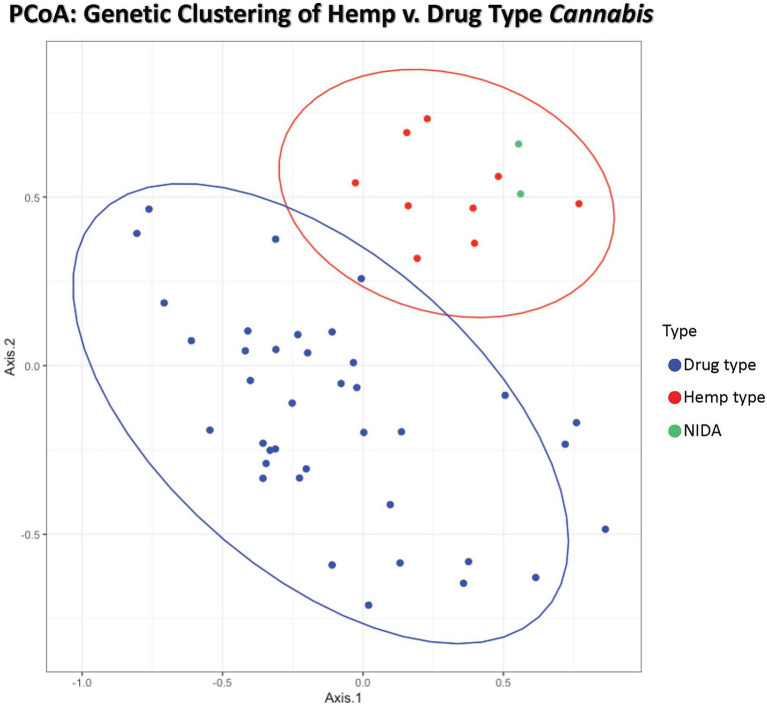
Principal coordinates analysis of genetic distance among samples. Samples clustering together are more closely related. The ellipses represent 95% confidence intervals for each group (Cultivated hemp = orange, Wild hemp = yellow, NIDA = blue, High CBD = pink, Sativa = red, Hybrid = green, Indica = purple). Approximately 24% of the genetic variation in these groups is shown (Axis 1 = 13.02% and Axis 2 = 11.17%).

**Figure 2:**
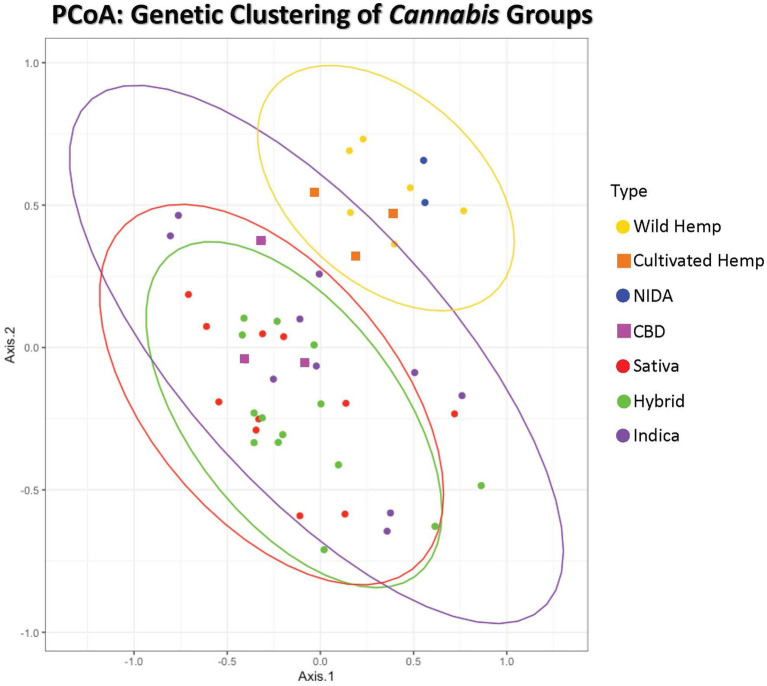
Principal Coordinates Analysis of genetic distance among samples. Samples clustering together are more closely related. The ellipses represent 95% confidence intervals for each group (Cultivated hemp = orange, Wild hemp = yellow, NIDA = blue, High CBD = pink, Sativa = red, Hybrid = green, Indica = purple). Approximately 24% of the genetic variation in these groups is shown (coordinate 1= 13.02% and coordinate 2 = 11.17%). No confidence intervals were drawn for NIDA, High CBD, or Cultivated Hemp samples due to the small sample size (n = 2, n = 3, and n = 3, respectively).

**Figure 3:**
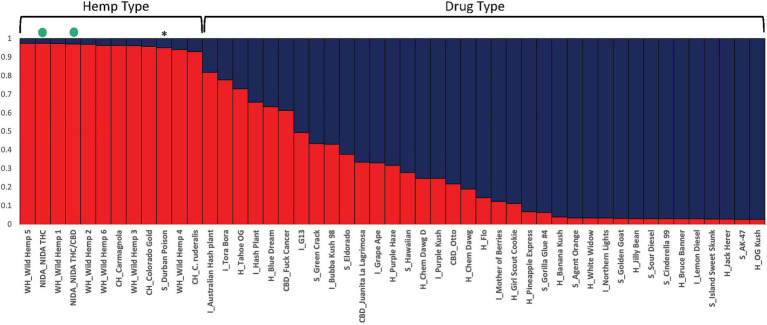
Bayesian clustering analysis from STRUCTURE with the proportion of inferred ancestry for two genetic groups (K=2) sorted by proportion of genotype assignment. Each individual is represented as a single bar in the graph. The NIDA samples are indicated by a green dot. * “Durban Poison” is a drug type assigned 0.95 to hemp ancestry. The letters preceding the sample name relate to the category the sample was place in (WH, wild hemp; CH, cultivated hemp; CBD, high CBD drug type; S, sativa drug type; H, hybrid drug type; I, Indica drug type).

**Figure 4:**
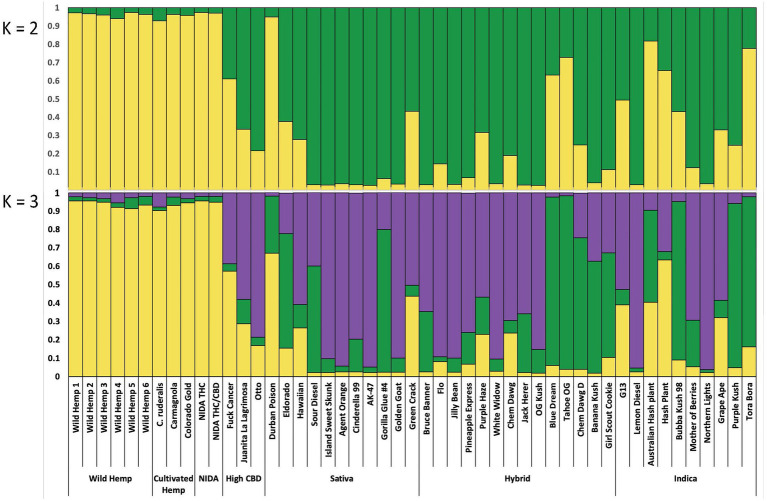
Bayesian clustering analysis from STRUCTURE with the proportion of inferred ancestry for two genetic groups (K=2, top), and for three genetic groups (K=3, bottom), Each individual is represented as a single bar in the graph.

Our analyses examined the genetic differentiation and structure of samples from seven *Cannabis* groups (Supplementary Table 1): (1) **Wild hemp** – feral wild collected hemp; (2) **Cultivated hemp** – obtained from hemp cultivators; (3) **NIDA** – “research grade marijuana” samples obtained from NIDA classified as high THC or high THC/CBD; (4) **high CBD** – drug-type *Cannabis* with relatively high levels of CBD and low levels of THC; and commercially available high THC drug-type *Cannabis* described as (5) **Sativa**, (6) **Hybrid**, or (7) **Indica**. With the exception of genetic distance statistics, the analyses were performed on samples at the individual level, where the genetic placement of each sample is determined independent of its’ putative *Cannabis* group. Conducting analyses at an individual level controls for biases that might arise due to the artificial nature of named groups and varying group sample sizes. Clustering (PCoA) and proportion of genetic assignment (STRUCTURE) analyses are presented first by assigning each sample by color to either hemp type or drug type (Figures 1, 2; Supplementary Figure 1), as these have previously been shown to separate well using genetic data (Datwyler and Weiblen, 2006; Piluzza et al., 2013; Sawler et al., 2015; Lynch et al., 2016; Dufresnes et al., 2017). The same analyses are then presented by color assignment to one of the seven subcategories to determine further possible relationships within and among these groups (Figures 3, 4).

### Genetic Analyses: Individual Level

#### Hemp V. Drug Types

Principal coordinate analysis (PCoA) with 95% confidence interval ellipses around the hemp-type (red) and drug-type (blue) groups shows clear separation of hemp samples from the drug types. NIDA samples are indicated in green and cluster within the hemp confidence interval (Figure 1). Coordinate 1 explains 13.02% of the genetic variation, and an additional 11.17% of the genetic variation is explained by coordinate 2.

STRUCTURE was used to examine sample assignment to genetic groups while allowing admixture. The appropriate number of STRUCTURE groups from K=1–10 was validated using STRUCTURE HARVESTER (Earl and vonHoldt, 2012), which had high support for two genetic groups (K=2, ∆K=61.35). An additional genetic structure analysis (MAVERICK 1.0.5: Verity and Nichols, 2016) was conducted to independently test group assignments and verified strong support for two genetic groups with the same assignment of individuals (K=2, probability 0.999, data not shown). The two genetic group STRUCTURE analysis (Figure 3) shows consistent differentiation between hemp-type and drug-type *Cannabis*. All hemp samples were assigned a genetic proportion of inferred ancestry (Q) greater than 0.92 (hemp mean group 1, Q=0.96). All but two drug-type samples showed admixture associated with hemp <0.78 (range 0.03–0.78) with 31 of 38 (83%) samples <0.50 proportion of ancestry associated with hemp genetic signal.

#### Categorical Group Analysis

Principal coordinate analysis with 95% confidence interval ellipses around the major groups shows that there is clear separation of hemp samples from the drug types, with NIDA samples (green) clustering within the hemp confidence interval (Figure 2). The drug-type samples (Indica, Sativa, Hybrid, and high CBD) all occupy the same character space, distinct from hemp.

For the categorical group STRUCTURE analyses, the two genetic group STRUCTURE analysis (K2, Figure 4) shows consistent differentiation between hemp- and drug-type samples. All hemp samples were assigned to genetic group 1 (yellow) with a proportion of inferred ancestry (Q) greater than 0.93 (hemp mean group 1, Q=0.96). High THC drug-type samples showed some admixture with 29 of 35 samples having the majority of the genetic signal assigned to genetic group 2 (green; high THC drug-type mean group 2, Q=0.75). The three high CBD drug-type samples were assigned with a mean of 0.61 to group 1 and 0.39 to groups 2. NIDA samples were assigned to genetic group 1 (NIDA mean group 1, Q=0.97), demonstrating a strong genetic association with hemp in this analysis.

Although not strongly supported, the three genetic group STRUCTURE analysis (K3, Figure 4) shows some additional genetic structure among drug-type samples. All hemp-type samples were assigned to genetic group 1 (yellow) with a proportion of inferred ancestry (Q) greater than 0.90 (hemp mean group 1, Q=0.93). The high THC drug-type samples demonstrated some admixture with 12 of 35 samples assigned genetic signal Q=>0.50 to group 2 (green; high THC drug-type mean group 2, Q=0.33), and 21 of 35 samples assigned genetic signal Q=>0.50 to group 3 (purple; high THC drug-type mean group 3, Q=0.53). The three high CBD drug-type samples were assigned with a mean of 0.34 to group 1, 0.10 to group 2 and 0.58 to group 3. NIDA samples were assigned to genetic group 1 (NIDA mean group 1, Q=0.95) with similarly low signal from groups 2 and 3 (0.03 and 0.02 respectively) demonstrating a strong genetic association with hemp. STRUCTURE analysis results are also presented from K=2–10 (Supplementary Figure 1).

### Genetic Analyses: Population Level

#### Genetic Differentiation

Pairwise genetic differentiation (Fst and Nei’s D) calculated in GENALEX ver. 6.4.1 [59, 60] found the highest level of divergence between NIDA and high CBD drug type (Fst=0.394) and between hemp and Sativa high THC drug type (Nei’s D=1.026; Table 1). The least divergence was observed among the high THC drug types (Fst=0.023–0.039; Nei’s D=0.066–0.102).

**Table 1:** Pairwise Fst values (below the diagonal) and Nei’s D (above the diagonal) for major *Cannabis* groups.

	NIDA	Wild Hemp	Cultivated Hemp	High CBD	Sativa	Hybrid	Indica
NIDA	-	0.738	1.018	0.911	1.026	0.918	0.808
Wild Hemp	0.245	-	0.386	0.500	0.606	0.605	0.475
Cultivated Hemp	0.324	0.086	-	0.532	0.652	0.614	0.518
High CBD	0.394	0.153	0.175	-	0.196	0.215	0.206
Sativa	0.319	0.117	0.143	0.092	-	0.098	0.102
Hybrid	0.310	0.122	0.147	0.096	0.039	-	0.066
Indica	0.268	0.083	0.109	0.092	0.033	0.023	-

#### Private Alleles

Private alleles, alleles found only in a single group, are commonly used in population genetic studies to identify divergent groups. Eight of the ten utilized loci contained at least one private allele in one *Cannabis* group (Table 2). Wild hemp contained the most private alleles, 12, while the high CBD group contained only 1. Given that we only sampled two NIDA individuals, the four observed private alleles indicate that this group contains unique genetic signal.

**Table 2:** Private alleles in each categorical group for ten loci. The number in parentheses after the locus name is number total number alleles for a locus.

	Total	Casa_02 (8)	Casa_06 (3)	Casa_14 (11)	Casa_18 (12)	Casa_22 (5)	Casa_26 (9)	Casa_27 (9)	Casa_28 (11)	Casa_29 (7)	Casa_30 (15)
NIDA	4				215		215		169		326
Wild Hemp	12	282		263			194, 239, 242	181	199	177, 180	267, 291, 294
Cultivated Hemp	3				203, 218				193		
High CBD	1	312									
Sativa	3						253	208			269
Hybrid	2			291	185						
Indica	3							187	196		297

## Discussion

The purpose of this study was to examine the genetic relationship of *Cannabis* samples from each of the common categories and subgroups and to determine where NIDA samples fall on the *Cannabis* genetic spectrum. The genetic regions used in this study were designed to target non-coding regions of the genome, and therefore less likely to reflect artifacts related to recent human selection. Our results clearly demonstrate that NIDA *Cannabis* samples are substantially genetically different from most commercially available drug-type strains and share a genetic affinity with hemp samples in several of the analyses. We do not claim that NIDA is supplying hemp for *Cannabis* research, rather we are confident that our analyses show that the “research grade marijuana” supplied by NIDA is genetically different from the retail drug-type samples analyzed in this study. Previous research has found that medical and recreational *Cannabis* from California, Colorado, and Washington differs significantly in cannabinoid levels from the “research grade marijuana” supplied by NIDA (Vergara et al., 2017). This investigation adds to the previous research, indicating that the sampled NIDA *Cannabis* is also genetically distinctive from commercially available medical and recreational *Cannabis*. Given both this genetic and previous chemotypic investigations have concluded that NIDA is supplying product that does not align with what is available for consumers, our hope is that the NIH and NIDA will support the cultivation of *Cannabis* that is representative of what medical and recreational consumers are using. Medical practitioners, researchers and patients deserve access to *Cannabis* products that are comparable to products available on the legal market.

The genetic data collected in this study indicate that two major genetic groups exist within *Cannabis sativa* (Figures 1, 3). These results contribute to the growing consensus that hemp- and drug-type *Cannabis* can be consistently differentiated (Forapani et al., 2001; Datwyler and Weiblen, 2006; McPartland, 2006; Hakki et al., 2007; Sawler et al., 2015; Lynch et al., 2016; Dufresnes et al., 2017; Soler et al., 2017), but all *Cannabis* groups are currently considered a single species that has been selected for different uses. Some admixture of the hemp-type genetic signal is seen in many of the drug-type samples; this is not unexpected as the legal definition of hemp (0.3% total THC by dry weight) is not biologically significant and therefore holds no scientific basis for formal taxonomic separation. To our knowledge, this study and collaborative work investigating the genomic *Cannabis* data (Vergara et al., 2021) are the first to include “research grade marijuana” from NIDA. The placement of NIDA samples with hemp in multiple analyses was unexpected. However, it is important to note that some drug-type samples (e.g., “Durban Poison,” Figure 3) are also placed in the hemp-type genetic group. This finding supports that although there are two distinct *Cannabis* genetic groups (hemp type and drug type), some strains within those groups have been selected to have the characteristics that we do not commonly associate with their specific genetic background. Crosses between hemp-type and drug-type strains may have been intentional, such as the recently developed high CBD drug strains that have low THC concentrations or the development of auto-flowering drug strains that flower as a function of age rather than photoperiod, which is a trait historically seen in some hemp varieties (Punja et al., 2017). Additionally, most *Cannabis* strains are a product of clandestine breeding in underground markets, so their presumed lineage may not match their actual genetic group. Hence, the finding that NIDA samples belong in the hemp-type genetic group in several analyses does not make these samples hemp, but it does demonstrate that they are different than the majority of drug-type *Cannabis* found in the marketplace.

Analyses were also conducted to examine how NIDA samples relate to traditionally recognized subgroups of *Cannabis*. It is important to note that some of the subgroups we assigned samples to are largely artificial and were based on information provided by online databases, which is the information that a recreational or medical consumer would have access to (Leafly, 2018; PotGuide.com, 2018; Wikileaf, 2018; Seedfinder, 2020). Although the categories Sativa, Indica and Hybrid are frequently used in the *Cannabis* industry and among consumers, researchers have yet to find consistent phenotypic and/or genotypic traits driving these widely referenced categories (Hillig, 2005; McPartland, 2017; McPartland and Guy, 2017; McPartland and Small, 2020). Given the high degree of intentional hybridization among drug-type *Cannabis*, it stands to reason that we would not see clear genetic separation among these categories. Additionally, the growing interest in *Cannabis* with alternative combinations of cannabinoids other than THC has led to increased breeding efforts between hemp and drug types, further diluting any historical genetic distinctions that might have existed. Therefore, we did not expect the seven groups we used here to resolve as genetically unique. The analyses of genetic distance (Table 1) and private alleles (Table 2) support that NIDA samples are substantially diverged from all other *Cannabis* groups, including hemp, and contain a unique genetic profile. The high CBD drug-type samples are genetically more divergent from the hemp group than the high THC drug-type groups, suggesting that these are hybrids of hemp-type and high THC drug-type *Cannabis*. Additionally, the high CBD drug-type samples and several drug-type samples are admixed with some genetic signal assigned to both hemp and drug groups. Given the intentional breeding of different *Cannabis* groups and the fact that hemp-type and drug-type *Cannabis* are defined by total THC content, a trait under selection, the lack of genetic support for many distinct groups is not surprising.

The University of Mississippi National Center for Natural Products Research (NCNPR) produces research grade drug-type *Cannabis* for NIDA. NCNPR does not provide variety or strain information when filling *Cannabis* orders, so it is unclear what is currently grown for federally funded *Cannabis* research. Our data suggest that the NIDA *Cannabis* analyzed in this study was sourced from a single strain or two very closely related strains within the NCNPR stock. Without additional information about NCNPR *Cannabis* production, it is difficult to know how many strains are provided for federally funded research using *Cannabis* from NIDA. This study included only two *Cannabis* samples from NIDA which limits what we can conclude about the breadth of genetic diversity contained in NIDA collections. The inclusion of additional NIDA samples would be beneficial, but additional sampling would in no way change the genotypes of the samples included in this study, which was supplied to researchers conducting federally approved *Cannabis* research. Although the sample size of NIDA samples could impact their placement in group-based analyses of genetic distance (Table 1), all other analyses were carried out at an individual level (Figures 1–4, and Supplemental Figure 1) to avoid this issue. The exact cause of the genetic distinction in NIDA samples cannot be determined, but many factors could play a role such as directional selection, inbreeding, sourcing of ancestral strains not currently represented in the commercial market, and/or cross-pollination from wild or cultivated hemp. It is our hope that this study will inspire further investigation of additional material supplied by NIDA.

Our study indicates the need for additional research and refinement of our understanding of *Cannabis* genetic structure and how those differences might impact *Cannabis* consumers. As the demand for medical *Cannabis* increases, it is important that research examining the threats and benefits of *Cannabis* use accurately reflects the experiences of the general public.

Given the rapidly changing landscape of *Cannabis* regulation and consumption (ProCon, 2018a,b), it is not surprising that commercially available *Cannabis* contains a diversity of genetic types. Commercially available *Cannabis* has come to market through non-traditional means leading to many inconsistencies. We have previously documented (Schwabe and McGlaughlin, 2019) that there is substantial genetic divergence among samples within named strains, which only exacerbates questions about the impacts of *Cannabis* consumption. These results emphasize the need to increase consistency within the *Cannabis* marketplace, and the need for “research grade marijuana” to accurately represent what is accessible to consumers.

This study highlights the genetic difference between “research grade marijuana” provided by NIDA and commercial *Cannabis* available to medical and recreational users. Hence, research conducted with NIDA *Cannabis* may not be indicative of the effects that consumers are experiencing. Additionally, research has demonstrated that *Cannabis* distributed by NIDA has lower levels of the principal medicinal cannabinoids (THC and CBD) and higher levels of the THC degradation product cannabinol (CBN; Vergara et al., 2017). Taken together, these results demonstrate the need for there to be a greater diversity of *Cannabis* available for medical research and that the genetic provenance of those samples to be established to fully understand the implications of results.

## Data Availability Statement

The original contributions presented in the study are included in the article/Supplementary Material, further inquiries can be directed to the corresponding authors.

## Author Contributions

AS conceived the project, collected the samples, conducted DNA extractions, designed and optimized microsatellite primers, compiled and analyzed the data, and drafted manuscript content. CH conducted DNA extractions, compiled and analyzed the data, and prepared the first draft of the manuscript. RH provided DNA from the NIDA samples. MM directed the project, provided some funding, and contributed to statistical analysis and manuscript revisions. All authors contributed to the article and approved the submitted version.

## Funding

The University of Northern Colorado Graduate Student Association and the Gerald Schmidt Memorial Biology Scholarship awarded grants providing partial funding for this project. Funding was also obtained from the University of Northern Colorado School of Biological Sciences. These funding sources did not play any roles in the development, design, execution, or analysis of this study, nor did they contribute to the writing of the manuscript and had no input in the decision to publish this research.

## Conflict of Interest

The authors declare that the research was conducted in the absence of any commercial or financial relationships that could be construed as a potential conflict of interest.

## Publisher’s Note

All claims expressed in this article are solely those of the authors and do not necessarily represent those of their affiliated organizations, or those of the publisher, the editors and the reviewers. Any product that may be evaluated in this article, or claim that may be made by its manufacturer, is not guaranteed or endorsed by the publisher.
